# N-Methyl-D-Aspartate Receptor-Dependent Denitrosylation of Neuronal Nitric Oxide Synthase Increase the Enzyme Activity

**DOI:** 10.1371/journal.pone.0052788

**Published:** 2012-12-28

**Authors:** Zhong-Wei Qu, Wan-Ying Miao, Shu-Qun Hu, Chong Li, Xing-Li Zhuo, Yan-Yan Zong, Yong-Ping Wu, Guang-Yi Zhang

**Affiliations:** 1 Research Center of Biochemistry and Molecular Biology; Jiangsu Key Laboratory of Brain Disease Bioinformation, Xuzhou Medical College, Xuzhou, Jiangsu, People’s Republic of China; 2 Jiangsu Key Laboratory of Anesthesiology, Xuzhou Medical College, Xuzhou, Jiangsu, People’s Republic of China; Albany Medical College, United States of America

## Abstract

Our laboratory once reported that neuronal nitric oxide synthase (nNOS) S-nitrosylation was decreased in rat hippocampus during cerebral ischemia-reperfusion, but the underlying mechanism was unclear. In this study, we show that nNOS activity is dynamically regulated by S-nitrosylation. We found that overexpressed nNOS in HEK293 (human embryonic kidney) cells could be S-nitrosylated by exogenous NO donor GSNO and which is associated with the enzyme activity decrease. Cys^331^, one of the zinc-tetrathiolate cysteines, was identified as the key site of nNOS S-nitrosylation. In addition, we also found that nNOS is highly S-nitrosylated in resting rat hippocampal neurons and the enzyme undergos denitrosylation during the process of rat brain ischemia/reperfusion. Intrestingly, the process of nNOS denitrosylation is coupling with the decrease of nNOS phosphorylation at Ser^847^, a site associated with nNOS activation. Further more, we document that nNOS denitrosylation could be suppressed by pretreatment of neurons with MK801, an antagonist of NMDAR, GSNO, EGTA, BAPTA, W-7, an inhibitor of calmodulin as well as TrxR1 antisense oligonucleotide (AS-ODN) respectively. Taken together, our data demonstrate that the denitrosylation of nNOS induced by calcium ion influx is a NMDAR-dependent process during the early stage of ischemia/reperfusion, which is majorly mediated by thioredoxin-1 (Trx1) system. nNOS dephosphorylation may be induced by the enzyme denitrosylation, which suggest that S-nitrosylation/denitrosylation of nNOS may be an important mechanism in regulating the enzyme activity.

## Introduction

Nitric oxide (NO) is synthesized by nitric oxide synthases (NOS) which has three known isoforms in mammals: neuronal NOS (nNOS), endothelial NOS (eNOS), and inducible NOS (iNOS). NO, as a reactive free radical gas, plays a vital role in regulating many physiological and pathophysiological processes [1], [2]. In addition to the classic activation of the c-GMP-dependent pathway, NO can also regulate many protein functions by means of a c-GMP-independent pathway, including those of the S-nitrosylating proteins [3], [4]. The S-nitrosylation of protein thiols is a redox–based post-translational modification that modulates protein function and activity [5]. Many studies have now suggested that either exogenous or endogenous NO can S-nitrosylate cysteine residues [6].

Both nNOS and iNOS are expressed in brain. iNOS is not normally present in healthy tissue, but is induced shortly after ischemia and contributes to secondary late-phase damage. nNOS is primarily expressed in neurons, where it mediates early neuronal injury [7]. As a constitutive 160-kDa protein as are the other NO synthase isoforms, nNOS functions as a dimer consisting of two identical monomers which link each other through a cysteine-complexed Zn^2+^ at the dimer interface [8]–[10]. The monomer can be functionally divided into two major domains: a C-terminal reductase domain and an N-terminal oxygenase domain, between which there is a calmodulin (CaM) binding domain which is vital to both the structure and function of the enzyme [2]. The phosphorylation of nNOS at Ser^847^ is induced by Ca^2+^/calmodulin-dependent kinase II (CaMK II) in the rat hippocampus following transient forebrain ischemia [11]–[13]. This decreases the activity of nNOS by inhibiting the binding of Ca^2+^/calmodulin. nNOSα harbors a PDZ domain at its N-terminus [14], through which postsynaptic density protein 95 (PSD-95) links nNOS with NMDAR to generate the NMDAR-PSD95-nNOS complex [15], [16]. The NMDAR-PSD95-nNOS complex couples the activation of NMDAR to the production of NO and mediates NMDAR-dependent excitotoxicity [17]. The excessive activation of glutamate receptors is the principle reason for the neuronal death that occurs in ischemic stroke as NMDAR has a higher calcium ion permeability than α-amino-3-hydroxy-5-methyl-4-isoxazole propionate receptor (AMPAR) or kainate receptor (KAR). NMDAR has thus been the focus of a number of studies that have screened for new therapeutic targets for stroke. Although NMDARs mediate ischemic brain damage, blocking these receptors to protect neuronal injury leads to severe side effects [18]–[20]. Targeting nNOS activity may therefore be a more effective therapeutic approach to the treatment of ischemic damage.

The Trx system, which comprises Trx proteins, TrxR proteins and NAPDH, is involved in a large number of cellular functions such as DNA synthesis, apoptosis and redox signaling, and also in the role of these pathways in a variety of diseases, and is regarded as having a critical role in the defense against oxidative stress [21]. In recent years, Trx proteins have been reported to have a number of other functions, particularly in the reduction of inter- and intramolecular disulfide bonds in proteins, and in the generation of oxidized peroxiredoxins, which disrupt organic hydroperoxides, H_2_O_2_, and peroxynitrite [22]. TrxR proteins, which reduce a broad range of substrates including an oxidized form of thioredoxin, can also directly reduce lipid hydroperoxides, dehydroascorbic and lipoic acids. Recently, the interplay between Trx and S-nitrosylation has becomes a research area of great interest.

Our previous studies have shown that the S-nitrosylation of nNOS is decreased during rat cerebral ischemia followed by reperfusion, but the functions and underlying mechanisms remained unclear. In our current study, we report that nNOS can be S-nitrosylated by the exogenous NO donor GSNO in HEK293 cells, and that the Cys^331^ residue is the key nitrosylation site. Our analyses also show that nNOS is S-nitrosylated in resting hippocampal CA1 and primary cortical neurons but undergoes denitrosylation induced by ischemia/reperfusion in hippocampal CA1 neurons or by OGD/reoxygenation in primary cortical neurons. We demonstrate that nNOS denitrosylation is induced by calcium ion influx via NMDAR during the early stages of reperfusion, which is a Ca^2+^/CaM-dependent process principally mediated by the Trx1 system, and that nNOS denitrosylation is coupled with an increase in the activity of this enzyme.

## Materials and Methods

### Antibody and Reagents

The following primary antibodies purchased from Santa Cruz Biotechnology (Santa Cruz, CA) were used in the experiments: rabbit polyclonal anti-nNOS antibody (sc-648), rabbit polyclonal anti-CaM antibody (sc-5537), rabbit polyclonal anti-TRXR1 antibody (sc-20147) and rabbit polyclonal anti-actin antibody (sc-10731). Rabbit polyclonal anti-p-nNOS antibody (Ser847, #834849) was obtained from Abcam Biotechnology (Cambridge, MA). A secondary goat anti-rabbit IgG antibody was used in our experiments and was bought from Sigma-Aldrich (St. Louis, MO). BCIP and NBT were obtained from Promega (Madison, WI). The ApopTag® peroxidase *in situ* apoptosis detection kit (S7100) was purchased from Chemicon International, Inc. (Temecula, CA). S-nitrosoglutathione (GSNO, #N4148), N-ethylmaleimide (NEM, E1271), (+)-5-Methyl-10,11-dihydro-5H-dibenzo [a,d] cyclohepten-5,10-imine maleate (MK801, #M107), dithiothreitol (DTT, #43817), BAPTA (A1076), and DNCB (#43817) were all acquired from Sigma-Aldrich. Auranofin (sc-202476) was purchased from Santa Cruz Biotechnology. W-7 (BML-CA320) was purchased from Enzo Life Sciences, Inc. (Enzo Life Sciences, NY). The TrxR1 antisense oligodeoxynucleotides (TrxR1 AS-ODNs), TrxR1 missense oligodeoxynucleotides (TrxR1 MS-ODNs), TrxR1 primers, and β-actin primers were synthesized by Sangon Biotech Co. Ltd. (Shanghai, China). TrxR1 siRNAs were designed and synthesized by GenePharma Co. Ltd. (Shanghai, China). The NOS activity assay kit (A014-2) was purchased from Nanjing Jiancheng Bioengineering Institute (Nanjing, China). All other chemicals were purchased from Sigma-Aldrich unless otherwise stated.

### Ischemic Model and Drug Treatments

The experimental procedures were conducted in accordance with local ethics legislation of JiangSu province regarding the use of animals for research purposes and the study was approved by the ethics committee of XuZhou Medical College. Experiments were designed to minimize the number of animals used and their suffering. Adult male Sprague–Dawley rats weighing 200–250 g were used (Shanghai Experimental Animal Center, Chinese Academy of Science, Shanghai, China). Animals were maintained under standard laboratory conditions under artificial 12 hours light/12 hours dark cycle.

Transient cerebral ischemia was induced by four-vessel occlusion (4-VO) as described previously [23]. Briefly, rats were anaesthetized with chloral hydrate (300 mg/kg, intraperitoneally) followed by permanent occlusion of both vertebral arteries by electrocautery and isolation of both carotid arteries. The animals were allowed to recover for 24 h and fasted overnight. The next day, both carotid arteries were occluded with aneurysm clips to induce cerebral ischemia. After 15 min of occlusion, the aneurysm clips were removed for reperfusion. Rats that lost their righting reflex within 30 s and whose pupils were dilated and unresponsive to light were selected for the experiments. The rectal temperature was maintained at 36.5–37.5°C during the ischemia and reperfusion process. An EEG was used to ensure isoelectricity after carotid artery occlusion. Sham controls were prepared using the same surgical procedures except that the carotid arteries were not occluded. The rats were injected intraperitoneally with MK801 (3 mg/kg) dissolved in 0.9% NaCl 30 min before preconditioning ischemia. GSNO (0.1 mg/kg) and DTT (60 µg/kg) dissolved in 0.9% NaCl were administered intracerebroventricularly (bregma: 1.5**mm lateral, 0.8 mm posterior, 3.5 mm deep) 30 min before preconditioning ischemia. BAPTA or W-7 dissolved in 10 µl 1% DMSO at a concentration of 10 mM and 40 µM, respectively, were administered intracerebroventricularly 30 min before preconditioning ischemia. Auranofin (0.6 µg) or DNCB (0.6 µg) dissolved in 10 µl 1% DMSO were also administered intracerebroventricularly 30 min before preconditioning ischemia. 10 nmol of TrxR1 AS-ODNs, 10 nmol of TrxR1 MS-ODNs in 10 µl TE buffer (10 mM Tris-HCl (pH 8.0), 1 mM EDTA) were given to the rats every 24 h for three days and the same dose of vehicle (TE buffer) was used in the controls.

### Cell Culture and OGD

Neurons from the cortices of fetal Sprague-Dawley rats (18 days gestation) were prepared as described previously [24]. Briefly, cortices were meticulously isolated in ice-cold high-glucose Dulbecco’s modified Eagle medium (h-DMEM, Gibco BRL, Grand Island, NY). Cortical cells were dissociated by trypsinization [0.25% (w/v) trypsin and 0.05% EDTA in Ca^2+^ and Mg^2+^ free Hanks balanced salt solution] at 37°C for 15 min, followed by gentle triturating in plating medium (h-DMEM supplemented with 10% fetal bovine serum and 10% horse serum; Gibco BRL). Cells were seeded onto poly-L-lysine coated wells (Sigma) and incubated at 37°C in a 5% CO_2_ atmosphere. After 3 h, the cells were incubated in neurobasal medium supplemented with B-27 (Gibco BRL) and 0.5 mM glutamine, and then half-replaced twice weekly. Cultures were used after 18 days in vitro. For OGD (oxygen-glucose deprivation), the cultured neurons was washed three times with glucose-free Earl’s balanced salt solution (EBSS) followed by replacement of the culture medium with EBSS. The cells were then placed in an anaerobic chamber filled with 5% CO_2_ and 95% N_2_ for 2 h. OGD was ended by switching back to normal culture conditions.

HEK293 cells and SH-SY5Y (human neuroblastoma) cells were cultured under an atmosphere of 5% CO_2_ at 37°C in h-DMEM supplemented with 10% fetal bovine serum. OGD of SH-SY5Y followed the same procedure to that used for the primary cortical neurons except that the time of the oxygen-glucose deprivation was extended to 5 h.

### Plasmid Construction, Transfection and Treatment of Neuronal Cultures with Oligonucleotides

The pME18S-nNOS plasmid was a gift from Professor Yasuo Watanabe (Department of Pharmacology and Ophthalmology, Nagoya University School of Medicine, Showa-ku, Nagoya, Japan). Mutants were constructed using the QuikChange II kit (Stratagene, La Jolla, CA). The primer sequences used for PCR-directed mutagenesis of nNOS Cys^326^ to Gly were: forward, 5′-CACTGGAAACGGGGGGCACAGAGCAC-3′; reverse, 5′-GTGCTCTGTGCCCCCCGTTTCCAGTG-3′. For the mutagenesis of nNOS Cys^331^ to Gly, the primer sequences were: forward, 5′-CAGAGCACATTGGCATGGGCTCGATC-3′; reverse, 5′-GATCGAGCCCATGCCAATGTGCTCTG-3′. In addition, the mutant nNOS^C326G^ plasmid was used as the template and the primers for mutagenesis of nNOS Cys^ 331^ to Gly were utilized to construct the double-mutant nNOS^C326G/C331G^. For mutagenesis of nNOS Ser^847^ to Ala, the primer sequences were: forward, 5′-CAAGGTCCGATTCAACGCCGTCTCCTCCTATTC-3′; reverse, 5′-GAATAGGAGGAGACGGCGTTGAATCGGACCTTG-3′. The mutants were confirmed by automated nucleotide sequencing (Shanghai Sangon Biological Engineering Technology & Services Co., Ltd, Shanghai, China).

The plasmids and TrxR1 siRNAs were transfected into HEK293 cells using lipofectamine 2000 (Invitrogen, Carlsbad, CA) in accordance with the manufacturer’s protocol. TrxR1 siRNAs were designed and synthesized by GenePharma Co. Ltd. (a pool of four sense strands against TrxR1 mRNA; GCAGCCAAAUUUGACAAGATT, GAGCAUGGUAUCAAGUUUATT, GGCGUGAAGAUCAAUGAAATT, and GUCCCAACGACUGUGUUUATT). The negative control used was a UUCUCCGAACGUGUCACGUTT sense strand. SH-SY5Y cells were transfected with siRNAs and harvested as described previously [25].

The treatment of neuronal cultures with oligonucleotides was carried out as described previously with some modifications [26]. Briefly, at day 14 of culture, rat cerebral cortical neurons were treated with 1 µM of TrxR1 AS-ODNs or MS-ODNs every 24 h for five days (TrxR1 AS-ODNs, 5′-AGGGGCATCTTTAGAGTCATT-3′; MS-ODNs, 5′-GTACTGGAACTGTGAAGTCTC-3′). The oligonucleotides used were glucosinolated and contained modifying derivatives to prolong their half-lives.

### nNOS Activity Assay

The activity of nNOS was determined using an assay kit from Nanjing Jiancheng Bioengineering Institute (Nanjing, China) in accordance with the manufacturer’s protocol and as described previously [27]. The principle of this assay system is that NO produced from the reaction of L-Arg and O_2_ can be reacted with nucleophilic material to form a colored complex. The optical density of the colored complex is measured at a wavelength of 530 nm and is used to calculate the nNOS activity, expressed as U/mg protein. One unit of nNOS activity was defined as the production of 1 nmol NO per second per milligram of protein.

### RT-PCR

SH-SY5Y cells transfected with siRNAs were harvested and total RNA was isolated by using an extraction kit (Promega, Madison, WI). To amplify TrxR1, the following primer pair was used: 5′ (sense) primer: 5′-GGCCAACAAAATCGGTGAACACATGGAAG-3′, and 3′ (antisense) primer: 5′-CGCCAGCAACACTGTGTTAAATTCGCCCT-3′. The primer pair used to amplify β-actin was 5′ (sense) primer: 5′-TCAGGTCATCACTATCGGCAAT-3′, 3′ (antisense) primer: 5′-AAAGAAAGGGTGTAAAACGCA-3′. After 30 amplification cycles, the products were mixed with loading buffer and analyzed on a 1.5% agarose gel.

### Sample Preparation

Rats were decapitated immediately after reperfusion for different periods and the hippocampal CA1 neurons were isolated and quickly frozen in liquid nitrogen. The hippocampi were homogenized in an ice-cold buffer containing 50 mM 3-(N-morpholino) propanesulphonic acid (MOPS) (Sigma; pH 7.4), 100 mM KCl, 320 mM sucrose, 50 mM NaF, 0.5 mM MgCl_2_, 1 mM EDTA, 1 mM EGTA, 1 mM Na_3_VO_4_ (Sigma), 20 mM sodium pyrophosphate, 20 mM β-phosphoglycerol, 1 mM p-nitrophenyl phosphate (PNPP), 1 mM benzamidine, 1 mM phenylmethylsulphonylfluoride (PMSF) and 5 mg/ml each of leupeptin, aprotinin and pepstatin A. The homogenates were centrifuged at 800 g for 10 min at 4°C and the supernatants were carefully collected. HEK293 cells, SH-SY5Y cells or cortical cells were washed three times with cold phosphate-buffered saline after treatment with different drugs, and then harvested from the culture flask by homogenization as described above followed by sonication. Protein concentrations were determined by the method of Lowry et al. Samples were stored at −80°C until use.

### Detection of nNOS S-Nitrosylation

S-nitrosylation of nNOS was detected using the biotin switch assay as described previously with some modifications [6]. Briefly, DTT-free samples were treated with methyl methanethiosulfonate (MMTS) buffer (250 mmol/L HEPES, pH 7.7, 10 mmol/L EDTA, 0.1 mol/L neocuproine, 2.5% SDS, 20 mmol/L MMTS), and then free thiols were blocked. Unreacted MMTS were removed by protein precipitation in acetone (−20°C). Sodium ascorbate was used to convert the cysteine residues that had been S-nitrosylated to free thiols, which were then biotinylated with biotin-hexyl pyridyldithiopropionamide (HPDP). Proteins were precipitated by cold acetone followed by resuspension in HENS buffer (250 mM Hepes, pH 7.7, 1 mM EDTA, 0.1 mM neocuproine, 1% sodium dodecyl sulfate [SDS]). Biotinylated proteins were precipitated with streptavidin–agarose (Sigma) and eluted from the beads with a solution containing 20 mM Hepes–NaOH, pH 7.7, 100 mM NaCl, 1 mM EDTA, and 100 mM β-mercaptoethanol.

### Immunoprecipitation and Immunoblot

For immunoprecipitation, tissue homogenates (each containing 400 µg of protein) were diluted fourfold with immunoprecipitation buffer (50 mM Hepes buffer (pH 7.4) containing 150 mM NaCl, 10% glycerol, 1% Triton X-100, 0.5% Nonidet P-40, 1 mM EDTA, 1 mM EGTA, 1 mM PMSF, and 1 mM Na_3_VO_4_. Samples were preincubated for 1 h with 20 µl of protein A-sepharose CL-4B (Amersham Biosciences, Uppsala, Sweden) at 4°C and centrifuged to remove proteins that had adhered non-specifically to the A-sepharose. The supernatants were incubated with 2 µg of primary antibodies overnight at 4°C. After the addition of A-sepharose, the mixture was incubated at 4°C for another 2 h and the samples were washed three times with immunoprecipitation buffer and the proteins were eluted by boiling at 100°C for 5 min in SDS-PAGE loading buffer.

Immunoblot analysis was carried out following 10% SDS-PAGE gel. Proteins were electrotransfered to nitrocellulose membrane (Amersham Biosciences, Buckinghamshire, UK). After blocking for 3 h in Tris-buffered saline with 0.1% Tween 20 (TBST) and 3% bovine serum albumin, membranes were incubated overnight at 4°C with primary antibodies in TBST containing 3% bovine serum albumin. Membranes were then washed and incubated with alkaline phosphatase-conjugated secondary antibodies in TBST for 2 h and developed using NBT/BCIP color substrate (Promega). The density of the bands on the membrane was scanned and analyzed with an image analyzer (LabWorks Software; UVP, Upland, CA).

### Histology and TUNEL Staining

Rats were perfusion-fixed with 4% paraformaldehyde in 0.1 M sodium phosphate buffer (pH 7.4) under anesthaesia after 3 or 5 days of ischemia/reperfusion. Brains were removed quickly and further fixed with the same fixation solution at 4°C overnight. Post-fixed brains were embedded by paraffin, followed by preparation of coronal sections 6 µm thick using a microtome. The paraffin embedded brain sections were deparaffinized with xylene and rehydrated by ethanol at graded concentrations of 100–70% (v/v), followed by washing with water. The sections were stained with 0.1% (w/v) cresyl violet and were examined with light microscopy and the number of surviving hippocampal CA1 pyramidal cells per 1 mm length was counted as the neuronal density. TUNEL staining was performed using an ApopTag® Peroxidase In Situ Apoptosis Detection Kit according to the manufacturer’s protocol with minor modifications. The paraffin-embedded coronal sections were deparaffinized and rehydrated, and then treated with protease K at 20 mg/ml for 15 min at room temperature. Sections were incubated with reaction buffer containing TdT enzyme and at 37°C for 1 h. After washing with stop/wash buffer, sections were treated with anti-digoxigenin conjugate for 30 min at room temperature and subsequently developed colour in peroxidase substrate. The nuclei were lightly counterstained with 0.5% methyl green.

### Data Analysis and Statistics

Values in this study are expressed as the mean ±S.D. and statistical analysis of the results was carried out using one-way analysis of the variance (ANOVA) followed by the Duncan’s new multiple range method or Newman-Keuls test. *P* values of <0.05 were considered significant.

## Results

### Exogenous NO S-nitrosylates nNOS and Decreases its Activity in HEK293 Cells

**Figure 1:**
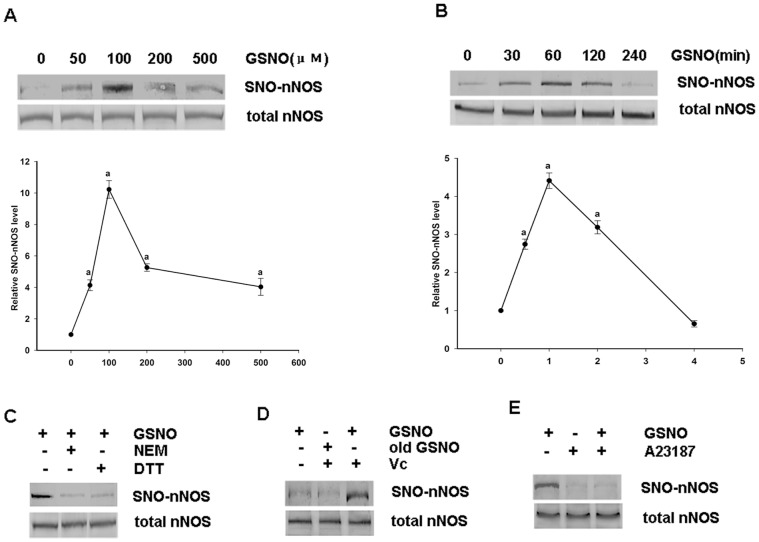
Effects of the exogenous NO donor GSNO on the S-nitrosylation of nNOS overexpressed in HEK293 cells. *A,* Optimization of the GSNO concentration required for nNOS S-nitrosylation. HEK293 cells were transfected with pME18S-nNOS for 48 h before treatment with different concentrations of GSNO for 1 h. *B,* Time course analysis of the SNO-nNOS levels in transfected HEK293 cells treated with GSNO (100 μΜ). *C,* Transfected HEK293 cells administrated with or without GSNO (100 μΜ), NEM (1 mM, a sulfydryl alkylating agent) or DTT (10 mM, a reducing agent). SNO-nNOS was then examined using a biotin-switch assay. *D,* Transfected HEK293 cells were administrated with GSNO or old GSNO, with or without Vc, during the biotin switch assay. *E,* transfected HEK293 cells were incubated for 30 minutes in the presence or absence of GSNO (100 μΜ) before treatment with or without A23187 (10 μΜ) for another 30 minutes. The bands were scanned and the intensities are expressed as the fold-changes with respect to the 0 group. The values shown are the mean ± SD (n = 4). ^a^ *P*<0.02 versus the 0 group.

To investigate the exogenous effects of NO on nNOS, HEK293 cells were transfected with wild type nNOS plasmid for 48 hours and treated with various concentrations of the NO donor GSNO for 60 min (Fig. 1A). Biotin switch assay was used to test the S-nitrosylation of nNOS. The results indicated that nNOS can be S-nitrosylated by exogenous NO and that 100 µM GSNO is the appropriate concentration to use in the reaction. We performed further time course analysis of GSNO treatments of nNOS to evaluate the optimal conditions and 1 hour was chosen (Fig. 1B). In addition, we found that when DTT (a reducing agent, dithiothreitol) was administrated 30 min after treatment with GSNO, the S-nitrosylation of nNOS was reversed. Similarly, when NEM (a sulfydryl alkylating agent) was used as a pretreatment to block the free thiols on proteins, S-nitrosylation was prevented (Fig. 1C). In contrast, GSNO exposed to light for at least 24 hours could not S-nitrosylate nNOS (Fig. 1D).

**Figure 2:**
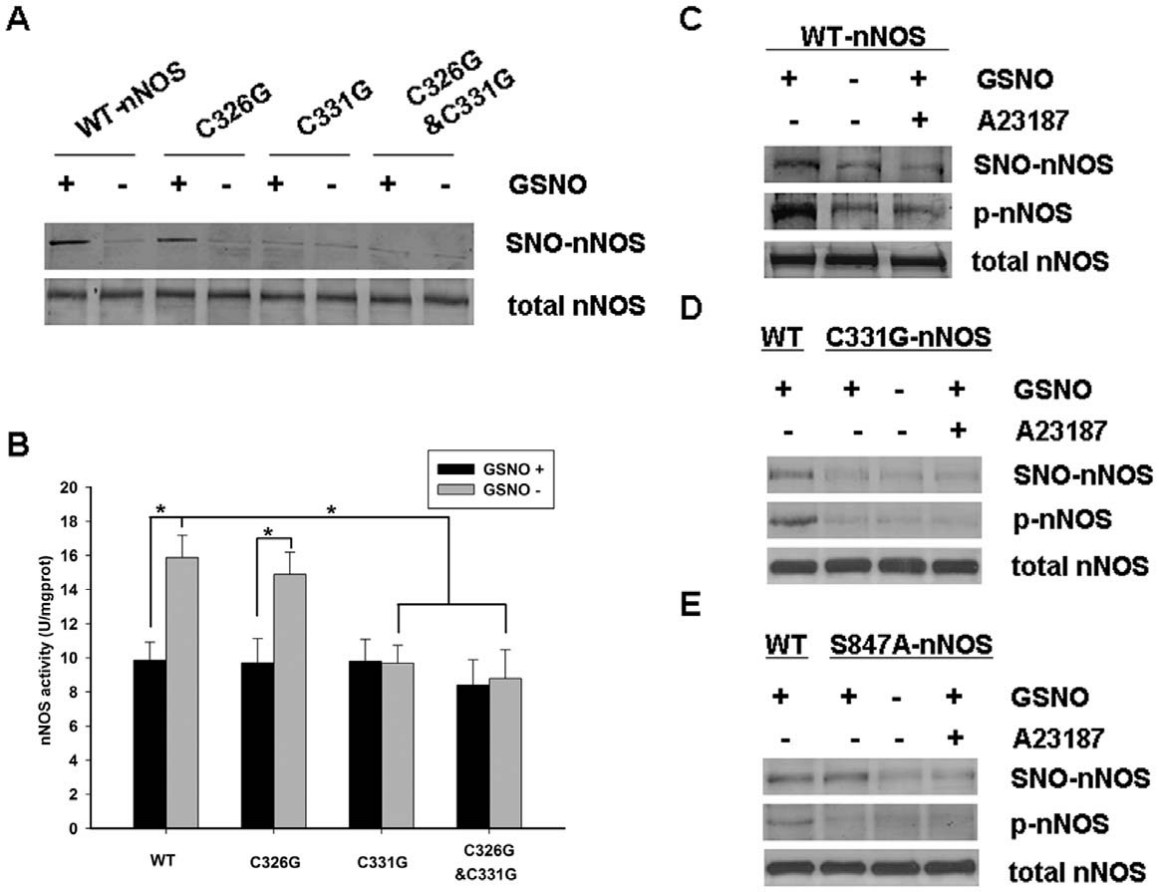
Identification of the nNOS S-nitrosylated site and examination of the regulation of nNOS S-nitrosylation. *A,* Wild type and mutant pME18S-nNOS plasmids were transfected into HEK293 cells for 48 hours, then the cells were then treated with GSNO (100 μΜ) or not for 1 hour. The blot shown is representative of three independent experiments that yielded equivalent results. *B,* Transfected HEK293 cells were incubated with or without GSNO and protein extracts were used to determine the nNOS activity levels. The enzyme activity levels were determined using the Nitric Oxide Synthase Kit (Nanjing Jiancheng Bioengineering Institute) in accordance with the manufacturer’s protocol. The data shown are the mean ± SD of five independent experiments (n = 5). ^*^ *P*<0.02 versus WT/C326G nNOS GSNO+ groups or WT nNOS GSNO- groups. *C, D and E*, Characterization of the S-nitrosylation and phosphorylation status of wild type nNOS (WT-nNOS), C331G nNOS and S847A nNOS exogenously expressed in HEK293 cells. The cells were incubated for 30 minutes in the presence or absence of GSNO (100μΜ) before treatment with or without A23187 (10μΜ) for an additional 30 minutes. The phosphorylation of nNOS was tested by immunoblotting with an anti-NP847 antibody. The blots shown are representative of three independent experiments.

As a negative control, was used the fact that nNOS cannot be S-nitrosylated if ascorbate is not administered during a biotin switch assay (Fig. 1D). The results showed that NO can bind to the free thiol of a protein when nNOS is S-nitrosylated. Because nNOS is a Ca^2+^/calmodulin-dependent enzyme, we used A23187, a calcium ionophore, to investigate the relationship between the S-nitrosylation of nNOS and Ca^2+^. We found that the S-nitrosylation of nNOS is significantly decreased by A23187 administered 30 min after GSNO treatment (Fig. 1E). To investigate the effects of exogenous NO treatment on nNOS activity, HEK293 cells transfected with wild type nNOS were treated for 1 hour with or without 100 µM GSNO. As shown in Figure 2A and 2B, GSNO attenuates nNOS activity.

### The Cys^331^Residue of nNOS is the Key S-nitrosylation Site

The zinc-tetrathiolate motif present in either nNOS or eNOS has been shown to be related to enzyme activity. The cysteines Cys^96^ and Cys^101^, which comprise the eNOS zinc-tetrathiolate complex, have been identified as the key sites of eNOS S-nitrosylation [28], [29]. We thus constructed and tested the nNOS mutants nNOS^C326G^, nNOS^C331G^ and nNOS^C326G/C331G^, in which the zinc-tetrathiolate complex cysteines were substituted for glycine. Wild type and mutant nNOS constructs were transfected into HEK293 cells and the biotin switch method was used to assay for S-nitrosylation. The results indicated that either wild type nNOS or nNOS^C326G^, but not nNOS^C331G^ or nNOS^C326G/C331G^ can still be S-nitrosylated by an exogenous NO donor (Fig. 2A). We further determined the activity of the different nNOS mutants compared with wild type nNOS, and found that the activity of nNOS^C331G^ and nNOS^C326G/C331G^ were significantly decreased whereas nNOS^C326G^ showed comparable activity levels (Fig. 2B).

### The Phosphorylation of nNOS is Regulated by S-nitrosylation

We next investigated the relationship between the phosphorylation and S-nitrosylation of nNOS. GSNO and A23187 were administered to cells transfected with WT-nNOS, C331G nNOS, and S847A NOS, respectively, and western blotting was used to test the levels of nNOS phosphorylation and S-nitrosylation. As shown in Figure 2C, when GSNO and A23187 were co-administered, the change in nNOS phosphorylation was consistent with the alteration in S-nitrosylation. When nNOS is S-nitrosylated by GSNO, the enzyme becomes phosphorylated. However, in the presence of A23187 the phosphorylation and S-nitrosylation of nNOS are decreased. We infer from these findings that a regulatory relationship exists between nNOS phosphorylation and S-nitrosylation. Interestingly however, the levels of S-nitrosylation and phosphorylation of nNOS^C331G^ were not altered after treatment with either GSNO or A23187 (Fig. 2D). This suggests that the phosphorylation of nNOS may be regulated by its S-nitrosylation status. The phosphorylation site of nNOS was then mutated (S847A nNOS, Fig. 2E) and S-nitrosylation of this mutant was found not to be influenced by any treatment, even though this variant cannot be phosphorylated. Taken together, we conclude from these data that the phosphorylation of nNOS is regulated by the extent of its S-nitrosylation and that this is a one-way process.

### NMDA Receptors Mediate nNOS Denitrosylation Induced by OGD/Rexoygenation or Cerebral Ischemia/Reperfusion and which Requires Ca^2+^ and CaM

**Figure 3:**
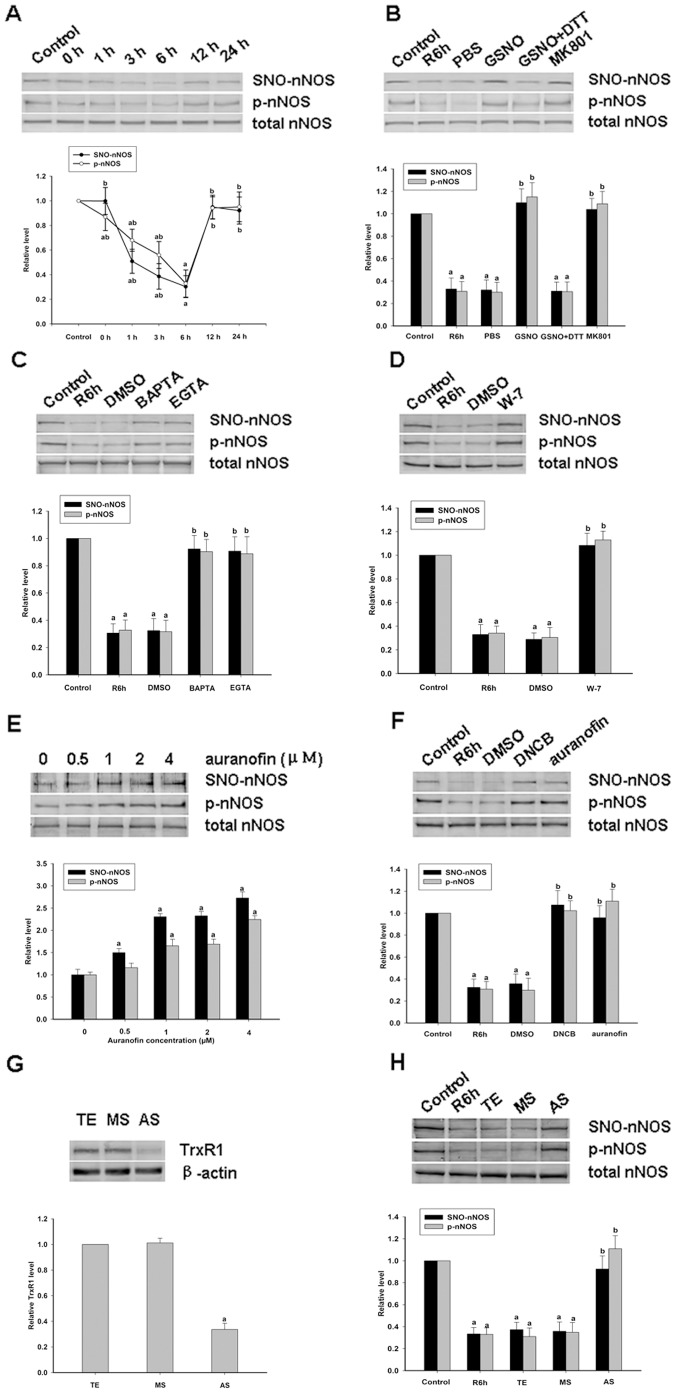
Effects of different drugs on the S-nitrosylation of nNOS in primary cortical neurons induced by oxygen-glucose deprivation/reperfusion (OGD/reoxygenation). *A,* Time course analysis of the S-nitrosylation and phosphorylation levels of nNOS in primary cortical neurons at various reperfusion time points after OGD. The data shown are the means ± S.D. from three independent experiments. ^a^ *P*<0.05 versus the control group; ^b^ *P*<0.05 versus the R6h group. *B,* The effects of GSNO (100 μΜ), or co-treatment with DTT (10 mM), and MK801 (20 μΜ) on the Ser847 phosphorylation and S-nitrosylation levels for nNOS induced by OGD followed by 6 hours of reoxygenation in primary cortical neurons. The blots shown are representative of three independent experiments (n = 3); ^a^ *P*<0.05 versus the control group; ^b^ *P*<0.05 versus the PBS group. *C,* Optimization of the auranofin concentration in the nNOS S-nitrosylation experiment in primary cortical neurons induced by OGD followed by 6 hours of reoxygenation. The bands shown are representative of three independent experiments. ^a^ *P*<0.05 versus the 0 μΜ group. *D-F,* The effects of auranofin (4 μΜ), DNCB (2 μΜ), BAPTA (4 μΜ), EGTA (25 mΜ) and W-7 (40 μΜ) on the Ser847 phosphorylation and S-nitrosylation of nNOS induced by OGD followed by 6 hours of reoxygenation in primary cortical neurons. The bands shown are representative of three independent experiments; ^a^ *P*<0.05 versus the control group; ^b^ *P*<0.05 versus the DMSO group. *G,* TrxR1 AS, TrxR1 MS oligonucleotide, or vehicle (TE) was administrated at a concentration of 1 µM to rat primary cortical neurons every 24 hours for five days prior to OGD. After 6 hours of reoxygenation, the cells were harvested and TrxR1 was analyzed by western blotting (n = 3); ^a^ *P*<0.05 versus the vehicle group. *H,* The effects of TrxR1 AS on the phosphorylation and S-nitrosylation of nNOS induced by OGD followed by 6 hours of reoxygenation in primary cortical neurons. The blots shown are representative of three independent experiments; ^a^ *P*<0.05 versus the control group; ^b^ *P*<0.05 versus the vehicle group.

To further confirm the relationship between nNOS denitrosylation and the enzyme activity, experiments were performed in cultured primary cortical neurons treated with OGD/reoxygenation. A time course analysis of reoxygenation was first performed to analyze changes in nNOS denitrosylation. We found that nNOS is S-nitrosylated in resting primary cortical neurons and undergoes significant denitrosylation after two hours of OGD with a peak reduction at 6 hours (Fig. 3A). nNOS is then subsequently renitrosylated to resting levels over an approximately over a 12 hours period. In parallel with these analyses, the phosphorylation of nNOS at Ser^847^, a site associated with nNOS activation, was also assessed. The results indicated that the S-nitrosylation of nNOS is related to its phosphorylation and activation.

**Figure 4:**
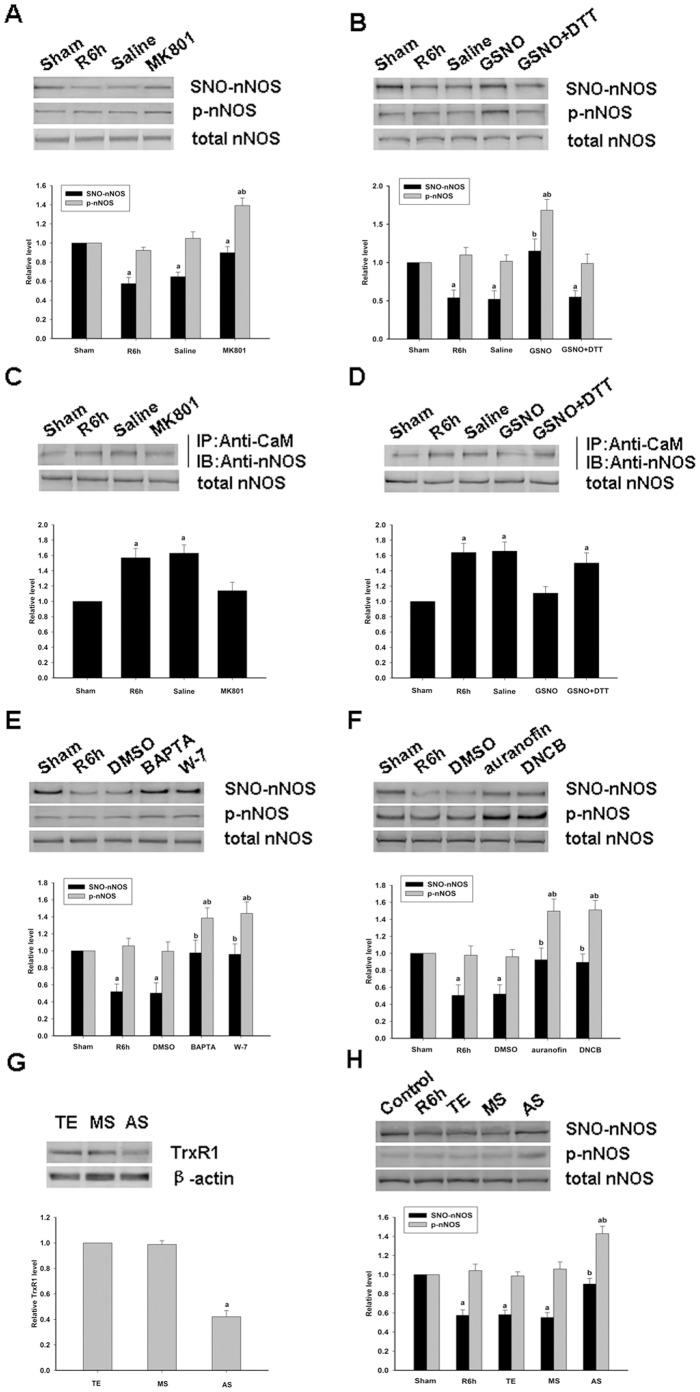
Effects of various drugs on the S-nitrosylation of nNOS in hippocampal CA1 neurons induced by cerebral ischemia/reperfusion. *A,* Western blotting analysis of the effects of MK801 on the Ser847 phosphorylation and the S-nitrosylation of nNOS induced by transient brain ischemia followed by 6 hours of reperfusion in rat hippocampal CA1 neurons. *B,* Western blotting analysis of the effects of GSNO, or its co-application with DTT, on the Ser847 phosphorylation and S-nitrosylation of nNOS induced by transient brain ischemia followed by 6 hours of reperfusion in rat hippocampal CA1 cells. *C-D,* Analysis of the effects of MK801, GSNO alone or with DTT, on the assembly of nNOS and CaM induced by transient brain ischemia followed by 6 hours of reperfusion in hippocampal CA1 cells. *E-F,* The effects of BAPTA, W-7, auranofin and DNCB, on the Ser847 phosphorylation and the S-nitrosylation of nNOS induced by transient brain ischemia followed by 6 hours of reperfusion in rat hippocampal CA1 neurons. *G,* 10 mmol of TrxR1 AS of TrxR1 MS oligonucleotides, or vehicle (TE) was administered to the rats every 24 hours for three days though a cerebral ventricular injection prior to cerebral ischemia. After 6 hours of reperfusion, protein extracts were made and TrxR1 was analyzed by western blotting. *H,* The effects of TrxR1 AS on the phosphorylation and S-nitrosylation of nNOS induced by cerebral ischemia followed by 6 hours of reperfusion in rat hippocampal CA1 cells. Sample proteins were examined by immunoprecipitation (IP) with anti-CaM antibody followed by immunoblotting with anti-nNOS antibody. The bands were scanned to calculate the intensities, which are represented as the fold-changes versus the sham treatment. The data values shown are the means±S.D. from four independent experiments; ^a^ *P*<0.05 versus the sham group; ^b^ *P*<0.05 versus the vehicle group.

There are a number of studies that have reported that protein denitrosylation can be induced by membrane receptors [30]. The NMDAR-PSD95-nNOS complex couples the activation of NMDAR with the production of NO and thereby mediates NMDAR-dependent excitotoxicity [18]. Hence, in our present study, the antagonist of NMDAR, MK801, was used to test the potential function of NMDAR in nNOS denitrosylation. Primary cortical neurons were treated with MK801 and as shown in Figure 3B, nNOS denitrosylation is suppressed when NMDARs are blocked by treatment with MK801, and the level of nNOS phosphorylation is increased. The effects of MK801 on the denitrosylation of nNOS in rat hippocampal CA1 neurons was next tested to further clarify the functions of the NMDA receptor and the results were similar to those obtained in primary cortical neurons (Fig. 4A). We thus examined the effects of GSNO on the denitrosylation of nNOS in rat hippocampal CA1 neurons and primary cortical neurons. The results showed that NOS denitrosylation was reversed by pretreatment with GSNO and abolished by co-treatment with DTT ((Fig. 3B and Fig. 4B).

Because nNOS is a Ca^2+^/CaM-dependent enzyme, we speculated whether Ca^2+^ and/or CaM is related to nNOS denitrosylation. Hence, we used MK801 and GSNO to treat rat hippocampal CA1 neurons and measured the effects of this on the assembly of nNOS and CaM. The results indicated that nNOS denitrosylation is coupled with the assembly of nNOS and CaM (Fig. 4C, D) which indicates that NMDAR mediates the denitrosylation of nNOS and is likely to be Ca^2+^/CaM-dependent. It has been demonstrated that Ca^2+^/CaM is vital to both the structure and function of the nNOS enzyme [2]. In our present experiments, we found that nNOS denitrosylation is coupled with the assembly of nNOS and CaM. To further investigate the possible relationship between the Ca^2+^/CaM complex and nNOS denitrosylation, and the underlying mechanism, we treated primary cortical neurons and rat hippocampal CA1 neurons, respectively, with W-7, EGTA and BAPTA. The results shown in Figures 3C, 3D and 4E show that pretreatment with both a Ca^2+^ chelator and CaM inhibitor prevents the nNOS denitrosylation and activation induced by OGD/reoxygenation or ischemia/reperfusion.

### The Trx1 System Mediates the SNO-nNOS Denitrosylation Induced by OGD/Reoxygenation or Ischemia/Reperfusion

Thioredoxin (Trx) systems have been suggested to mediate cysteine denitrosylation [30] and we speculated whether nNOS denitrosylation is also mediated by these pathways. Inhibitors of TrxR, DNCB and auranofin, which are rapid and efficient but dissimilar in structure, were tested for their effects on denitrosylation. First, an auranofin concentration course was performed using primary cortical neurons which were treated by reoxygenation six hours after OGD. As shown in Figure 3E, the treatment of primary cortical neurons with auranofin caused a dose-dependent inhibition of the decrease of S-nitrosylation and phosphorylation of nNOS induced by reoxygenation. Treatment of primary cortical neurons with DNCB, another inhibitor of TrxR, had similar effects (Fig. 3F). Identical results were obtained in rat hippocampal CA1 neurons induced by ischemia/reperfusion (Fig. 4F).

**Figure 5:**
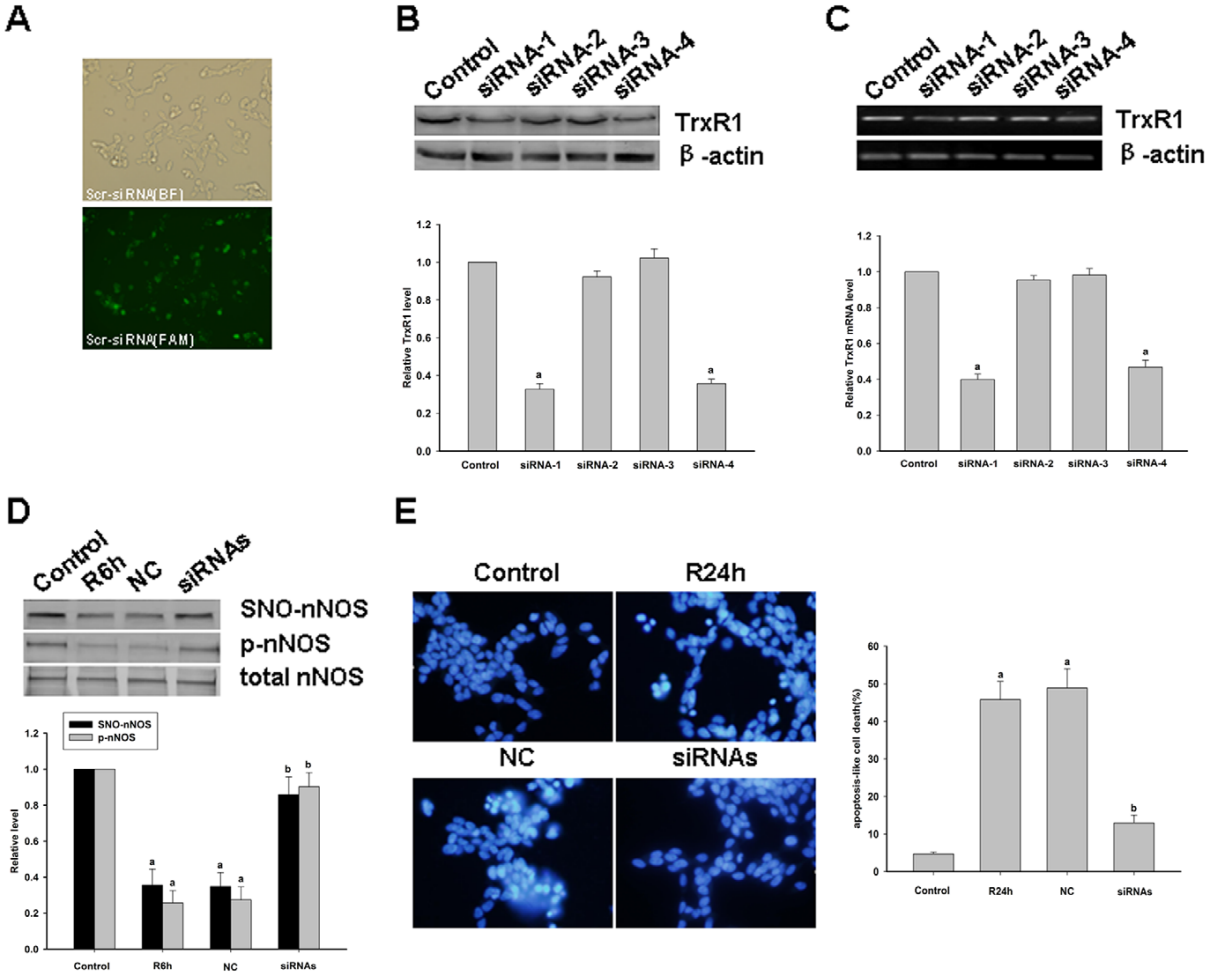
Effects of TrxR1 knockdown by siRNA in SH-SY5Y cells. *A,* Successful transfection of SH-SY5Y cells with a fluorescein-labeled scrambled-control siRNA. Photomicrographs were captured under a fluorescence microscope at a 400× original magnification at the end of the transfection period. BF, bright field; FAM, fluorescence from fluorescein-labeled scramble-siRNA. B, C, Relative protein expression and mRNA levels of TrxR1 are decreased in the siRNA-1 and siRNA-4 groups at 24 hours post-transfection as compared with the non-transfected control group. The results are the means±S.D. of three separate experiments; ^a^ *P*<0.05 versus the control group. D, The effects of an siRNA mixture (siRNA-1 and siRNA-4) on the S-nitrosylation and phosphorylation of nNOS in SH-SY5Y cells induced by OGD for 5 hours followed by 6 hours of reoxygenation. The bands shown are representative of three independent experiments; ^a^ *P*<0.05 versus the control group; ^b^ *P*<0.05 versus the negative control (NC) group. *E,* DAPI staining of SH-SY5Y cells induced by OGD for 5 h followed by 24 h of reoxygenation. Typical apoptotic cells with a condensed nucleus are indicated by arrowheads. Quantitative representations are expressed as a percentage of the total cells in 10 microscopic fields (×400) for DAPI staining. ^a^ *P*<0.05 versus the control group; ^b^ *P*<0.05 versus the NC group.

These findings suggest that nNOS denitrosylation induced by OGD/reoxygenation or by ischemia/reperfusion is indeed mediated by Trx systems. Trx systems can be divided into cytoplasm-specific (Trx1 system) and mitochondria-specific (Trx2 system) due to their subcellular location. Because nNOS is mostly present in the cytoplasm, we hypothesized that nNOS denitrosylation would be principally mediated by the Trx1 system. We employed siRNAs and AS-ODNs to deplete TrxR1 in SH-SY5Y cells, primary cortical neurons and rat hippocampal neurons and thereby further test the role of the Trx1 system in mediating SNO-nNOS denitrosylation. As shown in Figures 3H, 4H and 5D, nNOS denitrosylation was inhibited when the expression of TrxR1 was downregulated. These findings indicate that nNOS denitrosylation is indeed mediated mainly by the Trx1/TrxR1 systems.

### The Protective Effects of Some Drugs Against the Apoptosis Induced by OGD/reoxygenation or Ischemia/Reperfusion are exerted through the Prevention of nNOS denitrosylation

**Figure 6:**
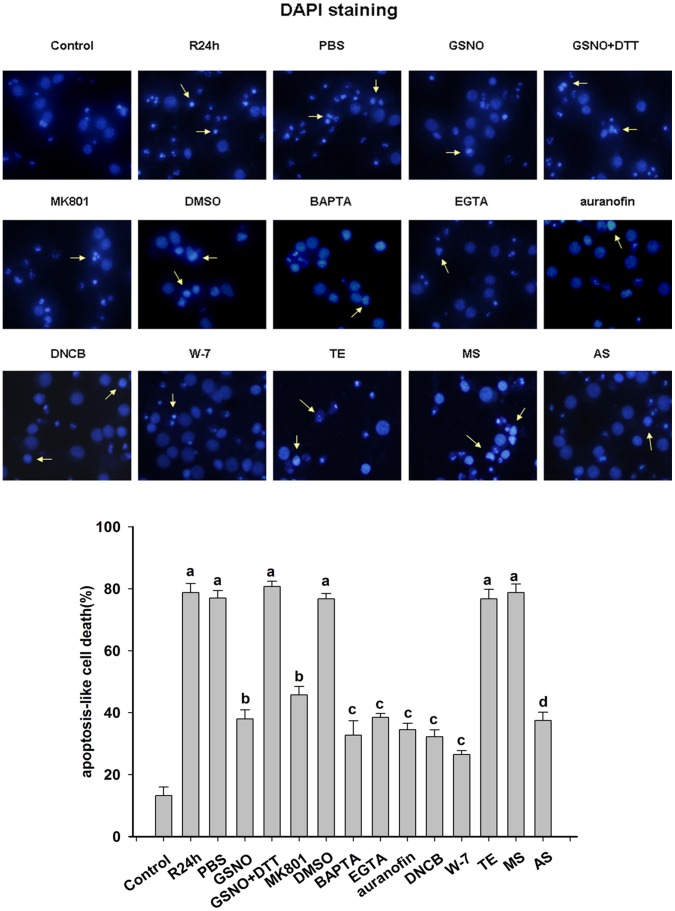
Inhibition of primary cortical neuronal apoptosis induced by OGD/reoxygenation. DAPI staining of primary cortical neurons was carried out at 24 hours after OGD. Typical apoptotic cells with condensed nuclei are marked by arrowheads. Primary cortical neurons of the R24h group were subjected to oxygen-glucose deprivation for two hours. Neurons in the drug treatment groups were exposed to GSNO, GSNO+DTT, MK801, BAPTA, EGTA, auranofin, DNCB and W-7 for 15 min before OGD. TrxR1 AS-ODNs or MS-ODNs were then administered every 24 hours for five days from day 14 of start of the cultures. PBS, DMSO and TE groups were used as vehicle controls. The number of apoptotic-like cells was expressed as a percentage of the total cell population counted in 10 microscopic fields (×400) via DAPI staining. The data shown are the means ± S.D. of four independent experiments;^ a^ *P*<0.05 versus the control group; ^b^ *P*<0.05 versus the PBS group; ^c^ *P*<0.05 versus the DMSO group; ^d^ *P*<0.05 versus the TE group.

Morphology experiments were performed to examine the survival of SH-SY5Y cells, primary cortical neurons and pyramial neurons of the rat hippocamal CA1 regions after various treatments. DAPI staining was used to determine the apoptotic levels of SH-SY5Y cells and primary cortical neurons. As shown in Fig. 5E and Fig. 6, the downregulation of TrxR1 by siRNAs or AS-ODNs significantly attenuated OGD/reoxygenation-induced neuronal apoptosis. In addition, MK801, GSNO, a Ca^2+^ chelator, an inhibitor of CaM and an inhibitor of TrxR were also found to protect against OGD/reoxygenation-induced neuronal apoptosis, indicating that nNOS denitrosylation is a key mechanism underlying the apoptotic response in primary cortical neurons or SH-SY5Y cells following OGD/reoxygenation.

**Figure 7:**
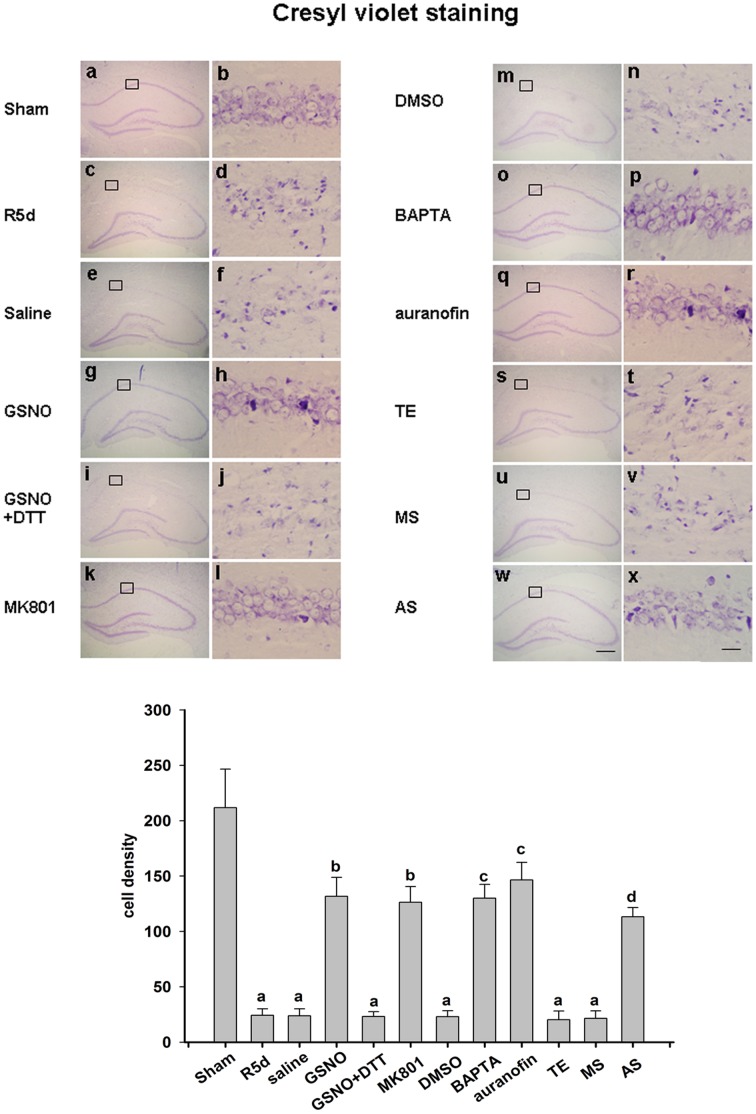
Neuroprotective properties of nNOS denitrosylation inhibition against neuronal injury in rat hippocampal CA1 cells induced by transient brain ischemia followed by five days of reperfusion. Coronal sections are shown from rats subjected to sham (panels a and b), R5d (panels c and d), pretreatment with saline (panels e and f), GSNO (panels g and h), GSNO+DTT (panels i and j), MK801 (panels k and l), DMSO (panels m and n), BAPTA (panels o and p), auranofin (panels q and r), TE (panels s and t), MS (panels u and v) and AS (panels w and x) treatments. Results were obtained from six independent animals from each experimental group, and the results of a typical experiment are presented. The boxed area in the left column is shown at a higher magnification in the right column. The original magnification is ×40 in all panels. Scale bars, 200 µm (panel f) and 10 µm (panel l). The numbers of viable cells were counted over a 1 mm region; ^a^ *P*<0.05 versus the sham group; ^b^ *P*<0.05 versus the saline group;^ c^ *P*<0.05 versus the DMSO group; ^d^ *p*<0.05 versus the TE group.

A similar experiment was performed in an animal ischemia model. As shown in Figure 7, cresyl violet staining was used to test the status of the neuronal cells in this model. Normal neurons show round and pale stained nuclei, as shown in the sham operation group, whereas shrunken cells with pyknotic nuclei were regarded as dead. As indicated in Figure 7 (panels c, d), 15 min of ischemia followed by five days of reperfusion led to severe cell death compared with the sham operation. Samples pretreated with GSNO showed dramatically decreased neuronal degeneration (Fig. 7, panels g, h), but this effect was abolished by co-treatment with DTT (Fig. 7, panels i, j). Treatment with MK801, BAPTA, auranofin and TrxR1 AS-ODNs exerted a neuroprotective effect against cerebral ischemia–reperfusion over the same time course (Fig. 7, panels k, l; o, p; q, r; w, x), whilst a saline control, DMSO control, TE control and MS-ODNs did not show such protective effects (Fig. 7, panels e, f; panels m, n; panels s, t; panels u, v).

**Figure 8:**
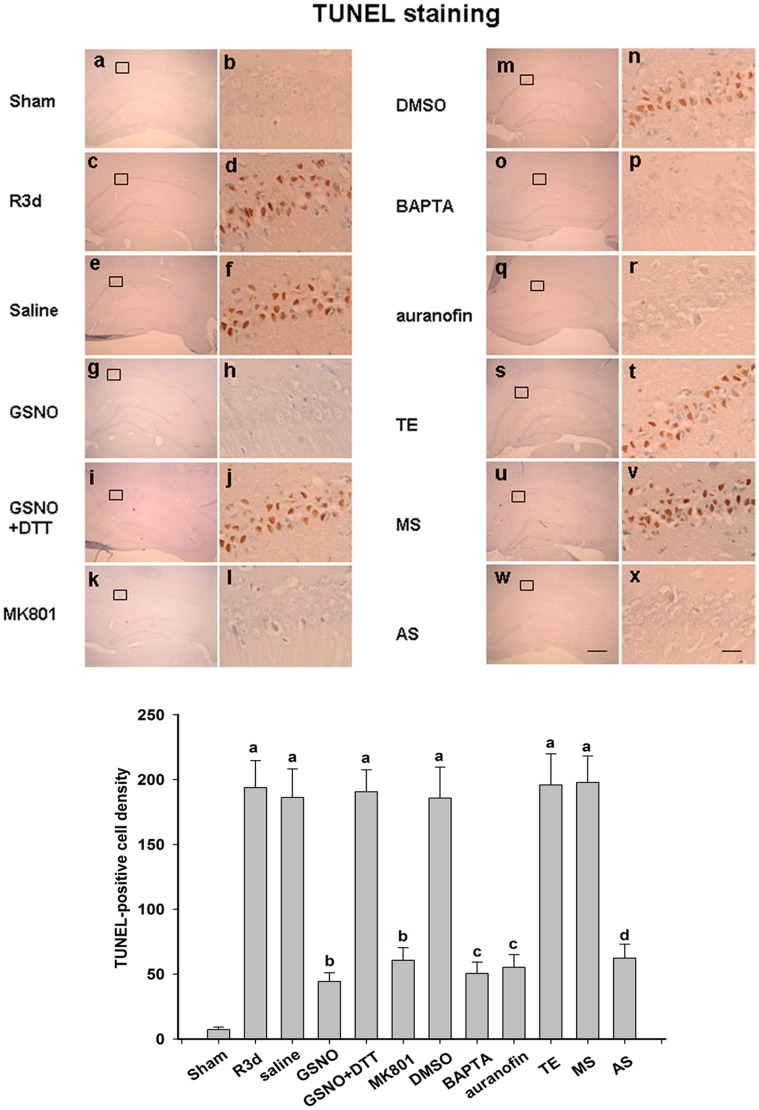
Representative photomicrographs of hippocampal cells subjected to TUNEL staining and counterstained with methyl green. Coronal sections are shown from rats subjected to sham (panels a and b), R3d (panels c and d), pretreatment with saline (panels e and f), GSNO (panels g and h), GSNO+DTT (panels i and j), MK801 (panels k and l), DMSO (panels m and n), BAPTA (panels o and p), auranofin (panels q and r), TE (panels s and t), MS (panels u and v) and AS (panels w and x) treatments. Results were obtained from six independent animals from each experimental group, and the results of a typical experiment are presented. The boxed area in the left column is shown at a higher magnification in the right column. The original magnification is ×40 in all panels. Scale bars, 200 µm (panel f) and 10 µm (panel l). The numbers of viable cells were counted over a 1 mm region; ^a^ *P*<0.05 versus the sham group; ^b^ *P*<0.05 versus the saline group; ^c^ *P*<0.05 versus the DMSO group; ^d^ *P*<0.05 versus the TE group.

TUNEL staining was also performed to examine the level of neuronal apoptosis in rat hippocamal CA1 regions. As shown in Figure 8, a significant number of TUNEL-positive cells were observed at three days after reperfusion (Fig. 8, panels c, d). Pretreatment with GSNO, MK801, BAPTA, auranofin or TrxR1 AS-ODNs clearly reduced the number of TUNEL-positive cells (Fig. 8, panels g, h; panels k, l; panels o, p; panels q, r; panels w, x). However, treatment with saline, the co-application of GSNO and DTT, and the administration of DMSO, TE or MS-ODNs, did not exert any protective effects (Fig. 8, panels e, f; panels i, j; panels m, n; panels s, t; panels u, v). Taken together, these results suggest that drugs that prevent nNOS denitrosylation can protect against neuronal apoptosis induced by OGD/reoxygenation in primary cortical neurons or by cerebral ischemia/reperfusion in hippocamal CA1 neurons.

## Discussion

In our present report, we demonstrate that overexpressed nNOS can be S-nitrosylated by the exogenous NO donor GSNO in HEK293 cells and that the activity of nNOS is thereby decreased. Cys^331^ was identified as the key site of nitrosylation on nNOS. Further, nNOS is highly S-nitrosylated in resting hippocampal CA1 and primary cortical neurons which was found to undergo denitrosylation induced by ischemia/reperfusion in hippocampal CA1 neurons or by OGD/reoxygenation in primary cortical neurons. Our current data demonstrate for the first time that nNOS denitrosylation is related to NMDAR, calcium, and CaM and is principally mediated by the Trx1 system. We further show that nNOS denitrosylation is coupled with increased activity of this enzyme, this process may be exerted by inducing the enzyme dephosphorylation. Moreover, the prevention of nNOS denitrosylation exerts protective effects on neurons by inhibiting apoptosis induced by both OGD/reoxygenation in primary cortical neurons and ischemia/reperfusion in hippocampal CA1 neurons.

S-nitrosylation and denitrosylation of protein cysteinyl residues have been suggested to be important NO-dependent post-translational modifications that in addition to phosphorylation and dephosphorylation can globally modulate protein function and activity both under physiological and pathological conditions [31]. It have been reported that many proteins can be S-nitrosylated by NO synthesized by nNOS. For example, GluR6 (glutamate receptor 6) is S-nitrosylated by nNOS-derived NO during the early stages of cerebral ischemia/reperfusion and induces neuronal apoptosis [32]. JNK3 (c-Jun N-terminal kinase 3), a pro-apoptosis kinase, is activated via S-nitrosylation induced by nNOS during cerebral ischemia/reperfusion in the same way [33]. MMP-9 (matrix metalloproteinase 9), a pro-apoptosis proteinase, is activated by S-nitrosylation induced by nNOS and induces neuronal apoptosis [34]. PTEN (phosphatase and tensin homolog deleted on chromosome 10) is also S-nitrosylated by nNOS and is involved in ischemic brain injury in the rat hippocampal CA1 region [35].

The evidence thus indicates that nNOS is a critical source of NO and thereby mediates protein S-nitrosylation. We wanted to determine however, whether the activity of nNOS itself is regulated in this way. Our previous study showed that nNOS is denitrosylated during cerebral ischemia and reperfusion in the rat hippocampus, but the underlying mechanism and functional significance of this remained to be elucidated [32]. It has been suggested that the activity of all three NOS enzyme isoforms can be inhibited through the formation of a complex between NO and the heme moiety of these proteins [36]–[39]. However, new findings indicate that the activity of eNOS can be inhibited by NO in another way i.e. S-nitrosylation at cysteine thiol groups that induces dimer disruption and dephosphorylation [28], [29]. Hence, the aim of our present study was to test whether the activity of nNOS is also regulated by S-nitrosylation/denitrosylation.

Our present results reveal that the S-nitrosylation of nNOS leads to decrease in enzyme activity through an analysis of the effects of the physiological NO donor GSNO on the activity and S-nitrosylation of nNOS overexpressed in HEK293 cells. As shown in Figure 2A and 2B, nNOS S-nitrosylation is inversely related to its enzyme activity levels in vitro. Furthermore, the S-nitrosylation of nNOS and the inhibitory effects on its enzyme activity induced by NO can be reversed or blocked by ascorbate, DTT and NEM, respectively (Fig. 1C and 1D). Our results further indicate that S-nitrosylation occurs on cysteinyl residues and can be removed by reducing agents in parallel with denitrosylation. Our data thus suggest that the inhibitory mechanism of S-nitrosylation on enzyme activity may have important physiological roles in vivo.

An important question arising from our present analyses is how nNOS enzyme function is actually regulated by S-nitrosylation of a cysteine residue. Cysteine residues can be involved in regulating protein activity and in signaling events through reactions involving their thiol groups, including redox events (chemical reduction or oxidation), the chelation of transition metals (chiefly Zn^2+^, Mn^2+^ and Cu^2+^) or S-nitrosylation [40]. It has been reported that the Cys^99^ residue of human eNOS is responsible for tetrahydrobiopterin (BH_4_) binding [41]. Recently also, Cys^99^ of the eNOS tetrathiolate cluster was found to be S-nitrosylated by an NO donor and thought to alter pterin binding, mediate the loss of Zn^2+^ and convert the eNOS dimer into a monomer, with a resultant loss of enzyme activity [28]. In contrast, another study concludes that the S-nitrosylation of eNOS alters its enzyme activity in association with changes in its phosphorylation and subcellular targeting status rather than disruption of its dimer form [29]. It has been reported that the C331A mutant of nNOS is defective in arginine binding and catalytic activity, and also that the imidazole binding affinity, CaM binding affinity, rates of cytochrome *c* and 2,6-dichlorophenolindophenol reduction in the C331A mutant are altered compared with the wild type nNOS protein [42]. We found in our current study that when the Cys^331^ residue (a critical site for Zn^2+^ binding) of nNOS was substituted by glycine, the S-nitrosylation of the protein and enzyme activity levels were significantly decreased (Fig. 2B). Based on these findings and the evidence of previous reports, we deduce that the regulation of nNOS activity via the S-nitrosylation of Cys^331^ is possibly exerted through the altered binding of cofactors such as Zn^2+^. More studies will be needed to properly elucidate this mechanism.

Given that the activity of nNOS is probably regulated by S-nitrosylation and it is well established that the phosphorylation of nNOS reduces its activity [43]–[45], we speculated as to which of these modifications is responsible for the activation of this enzyme. From the results of our OGD/reoxygenation time course experiment (Fig. 3A), we observed that nNOS is S-nitrosylated in resting primary cortical neurons. After treatment of these cells with OGD/reoxygenation, nNOS is denitrosylated to at the maximum extent at 6 hours and is then progressively renitrosylated to its original resting level at about 12 hours. Interestingly, the time course of nNOS phosphorylation at Ser^847^ accords with its observed S-nitrosylation pattern. Furthermore, through our analysis of GSNO, DTT, auranofin (inhibitor of TrxR) as well as DNCB (Fig. 3B, F), which alter the S-nitrosylation of nNOS, we found that nNOS phosphorylation was modulated at the same time. From the results described above, we conclude that nNOS phosphorylation has a consanguineous relationship with nNOS S-nitrosylation and that the enzyme is much more likely to be modulated by nNOS S-nitrosylation. Which raises one interesting question: what is the relationship between nNOS S-nitrosylation and phosphorylation? To investigate the relationship between the phosphorylation and S-nitrosylation of nNOS, the GSNO and A23187 were treated on the cells transfected with WT-nNOS, C331G nNOS, and S847A nNOS, respectively. As shown in Figures 2C, D, E, we speculate that the phosphorylation of nNOS was regulated by the enzyme S-nitrosylation and it is a one-way process. We remain uncertain of the precise mechanisms underlying this and further studies are planned in our laboratory to elucidate this.

Our previous experiments have demonstrated that nNOS S-nitrosylation and phosphorylation are significantly decreased during cerebral ischemia-reperfusion in the rat hippocampus CA1 region [35]. Interestingly, our present results show that there is no significant change between phosphorylation of nNOS at 6 hours and 12 hours of reperfusion compared with the sham controls. However, our current data indicate that nNOS S-nitrosylation is dramatically decreased at the same time points of reperfusion. Although both nNOS and iNOS are known to be expressed in brain, it is reported that only nNOS is continuously activated and produces NO during the early stages of cerebral ischemia, whilst iNOS protein expression and catalytic activity is detectable at 12 hours after cerebral ischemia and produces an excessive amount of NO in the late stages of ischemia [46]–[48]. Based on the above findings, 6 hours of reperfusion was chosen as the optimal condition to prevent the interruption of NO induction by iNOS.

Although a number of mechanisms of protein S-nitrosylation have been clarified previously, the mechanism of protein denitrosylation remains largely unknown. It is reported that calcium mediates denitrosylation [49] and as nNOS is a Ca^2+^/CaM-dependent enzyme we speculated as to whether its denitrosylation had any relationship with calcium and CaM. From the results shown in Figure 1E, Figure 3C and Figure 4E, we observe that nNOS denitrosylation can be induced by a calcium ion influx. Additionally, it has been reported that protein denitrosylation can be induced by membrane receptors [30]. The NMDAR-PSD95-nNOS complex couples the activation of NMDAR with the production of NO, which then mediates NMDAR-dependent excitotoxicity [17]. Furthermore, NMDAR has a higher calcium ion permeability than either the AMPAR or KAR. We therefore used MK801 in our current experiments and the results indicate that the calcium induced denitrosylation of nNOS is possibly mediated through NMDAR (Fig. 3B and Fig. 4A) during the process of ischemia-reperfusion or OGD/reoxygenation. Moreover, from the results showing that nNOS denitrosylation can be blocked by calcium chelators and a calmodulin inhibitor, we conclude that nNOS denitrosylation induced by OGD/reoxygenation or cerebral ischemia/reperfusion is dependent on the binding of the Ca^2+^/CaM complex.

Recently, several denitrosylases have been identified, such as S-nitrosoglutathione reductase, thioredoxin systems, protein disulphide isomerase (PDI), and xanthine oxidase (XO) [50], [51]. However, two cellular enzyme systems in particular have emerged as physiologically relevant denitrosylases [30], the S-nitrosoglutathione reductase (GSNOR) system, which comprises GSH and GSNOR, and the thioredoxin (Trx) system, which comprises Trx protein, Trx reductase (TrxR) and NADPA. The Trx system is present in all living organisms [52] and can be divided into cytoplasm-specific (Trx1 system) and mitochondria-specific (Trx2 system) by means of its subcellular localization. Because nNOS is predominantly present in the cytoplasm, we hypothesized that its denitrosylation would be principally mediated by the Trx1 system. First, auranofin and DNCB (inhibitors of TrxR), both rapid and efficient inhibitors but with dissimilar structures, were administered to cells and the denitrosylation of nNOS was inhibited (Fig. 3F and Fig. 4F). Next, siRNAs against TrxR1 and also TrxR1 AS-ODNs were designed to verify the function of the Trx1 system in inducing nNOS denitrosylation. The results indicate that nNOS denitrosylation is mainly mediated by the Trx1 system. Based on these results above, we conclude that the denitrosylation of nNOS is associated with calcium and the Trx1/TrxR1 system. However, more studies are required to elucidate the precise interactions between calcium, nNOS and Trx1 system. In addition, we cannot exclude the possibility that other denitrosylases are involved in this process.

Taken together, we reveal from our current data that Ca^2+^ and the Trx1 system are involved in the process of nNOS denitrosylation in a rat ischemia model. However, whether Ca^2+^ is related to the activity of the Trx1 system during this process is unclear. It has been reported that the expression and activity of TrxR1 is increased in human endothelial cells after treatment with the calcium ionophore A23187 [53]–[55]. It would be of interest therefore to measure the activity of TrxR1 and elucidate its possible relationship with Ca^2+^ during the process of ischemia.

In summary, our present data show that the activity and phosphorylation of nNOS are regulated by the S-nitrosylation of its zinc-tetrathiolate cysteine. The activity level of this enzyme is regulated by its S-nitrosylation level. Moreover, nNOS denitrosylation is principally mediated by calcium and the Trx1 system. nNOS activity is known to be regulated by complex post-translational modifications and protein-protein interactions. S-nitrosylation must now be included as a key nNOS post-translational modification which may have vital significance for the regulation of nNOS activity in the nervous system.
